# Childhood Obesity: A Narrative Review

**DOI:** 10.7759/cureus.82233

**Published:** 2025-04-14

**Authors:** Susmita Sinha, Rahnuma Ahmad, Kona Chowdhury, Shamima Islam, Miral Mehta, Mainul Haque

**Affiliations:** 1 Physiology, Enam Medical College and Hospital, Dhaka, BGD; 2 Physiology, Medical College for Women and Hospital, Dhaka, BGD; 3 Pediatrics, Enam Medical College and Hospital, Dhaka, BGD; 4 Forensic Medicine, Enam Medical College and Hospital, Dhaka, BGD; 5 Pedodontics and Preventive Dentistry, Karnavati School of Dentistry, Karnavati University, Gandhinagar, IND; 6 Pharmacology and Therapeutics, National Defence University of Malaysia, Kuala Lumpur, MYS; 7 Research, Karnavati School of Dentistry, Karnavati University, Gandhinagar, IND

**Keywords:** childhood obesity, heart disease, insulin resistance, low-income countries, metabolic syndrome, obesity, physical activity, psychosocial health, public health, stress

## Abstract

Obesity among children has emerged as a worldwide health issue due to childhood obesity becoming a pandemic, and it is often linked to various illnesses, fatal outcomes, and disability in adulthood. Obesity has become an epidemic issue in both developed and developing countries, particularly among youngsters. The most common factors contributing to non-communicable diseases (NCDs) are unhealthy eating habits, desk-bound games, avoidance of physical activity-requiring activities, smoking, alcohol usage, and other added items. All these factors increase NCDs, including obesity, resulting in various morbidities and early death. Additionally, childhood obesity has psychological, emotional, cognitive, societal, and communicative effects. For example, it raises the possibility of issues related to physical appearance, self-esteem, confidence level, feelings of isolation, social disengagement, stigma, depression, and a sense of inequality. Children who consume more energy-dense, high-fat, low-fiber-containing food than they need usually store the excess as body fat. Standardizing indicators and terminology for obesity-related metrics is critical for better understanding the comparability of obesity prevalence and program effectiveness within and between countries. The underlying variables must be altered to reduce or avoid harm to the target organ in children. As a result, reducing childhood obesity is a considerable public health goal for the benefit of society and the long-term well-being of individuals.

## Introduction and background

Perpetual nutritional scarcities illustrate an expeditious dietary nourishment and epidemiological conversion globally because of altered dietary patterns, the increased incidence of micronutrient deficiency as substantiated by children's low growth rate, increased possibility of acute infectious disease, and many pathological issues, or even fatal outcome [1-4]. Concurrently, there is a continuing escalation in the frequency of nutrition-related chronic diseases (NRCDs) and non-communicable diseases (NCDs), diabetes mellitus, overweight, obesity, cardiovascular disease, metabolic syndrome (MetS), orthopedic, neurological, hepatic, pulmonary, renal disorders and certain types of carcinomas [5-7]. The World Health Organization (WHO) reported that more than 0.39 billion children and teenagers aged 5-19 years were overweight in 2022; among them, 0.160 billion people suffer from respiratory problems due to obesity [8]. Multiple studies reported that childhood obesity is the most puzzling and complicated public health issue in the 21st era and initiating intense insinuations for equal physical and mental health [9,10]. Childhood obesity is a global health problem due to its pandemic-like appearance. It is frequently linked to multiple morbidities, fatal outcomes (increased possibility of premature death before the age of 30), and infirmity in adulthood [9,11,12]. Regular consumption of high energy-containing foods, e.g., sugar-sweetened beverages (SSBs) [13-15], fast foods [16-18], baked greasy foods [19,20], vending machine snacks [21,22], and overall ready-to-eat meals [23,24], causes children to gain weight. Multiple studies reported that obesity issues reach has been reached to extreme health issue, especially among children and women in high-income countries (HICs) [25-27]; however, issues of obesity and patterns of diet alternation between low- and middle-income countries (LMICs) have reached an alarming level because increased availability convenience store meal and snacks [2,28-30].

In childhood and adolescence, obesity habitually endures into man or womanhood [31,32], causing both feelings of aloneness or social seclusion [33], with high proportions of multiple concomitant diseases [34] such as cardiovascular, liver disease, insulin resistance (IR), type 2 diabetes mellitus (T2DM), bronchial asthma, exercise intolerance, sleep apnea (sleep-disordered breathing), and MetS [35-40]. Health promotional activity is essential to maintaining a good quality healthy life and should start in early childhood to prevent precipitating factors for NCDs [41]. The most dominant feature that causes NCDs are unhealthy food consumption habits, desk-bound gaming, avoidance of outdoor play that requires physical activity, habituation of smoking, alcohol drinking, and other addictive products [42]. All these factors promote NCDs, including obesity, thereby ensuring multiple morbidities and premature death [43-46]. Primary health care (PHC) can eventually prevent NCDs among children by early diagnosis, prevention, and appropriate therapeutic management in a nascent stage [47,48]. ﻿WHO endorses three components of PHC that need to be implemented for unfolding and installing an effective response to freeze and drive back the escalating trail of obesity globally: "integrated health services, multisectoral policy and action, and empowered people and communities" [49].

Problem statement of this review paper

**Figure 1:**
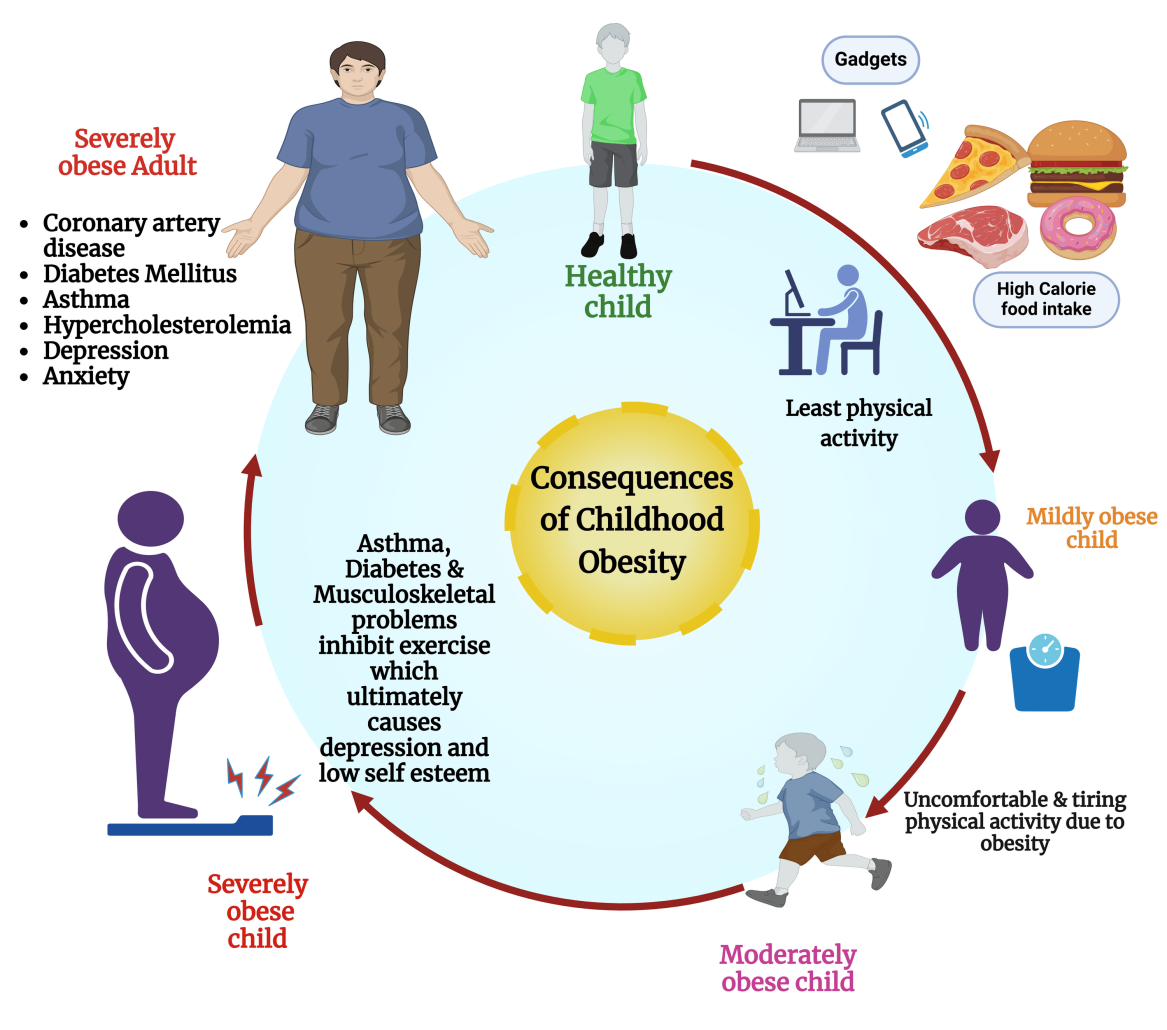
Consequences of childhood obesity. Notes: This figure was drawn using the premium version of BioRender (https://biorender.com/wpxxuld) [59], which was accessed on 27 March 2025, with license number RA282OORX6. Illustration Credit: Susmita Sinha.

Globally, childhood obesity is a grave public health concern [50]. Leung et al. (2024) reported that juvenile overweightness, when dyed in the wool (fixed), is habitually noncompliant with therapeutic intervention. Most beneficial strategies for weight loss frequently exist for a short time. The lost weight started regaining after stoppage strategies were adopted [51]. Childhood obesity often leads to life-threatening health disorders (Figure 1), e.g., increases the risk of hypertension, high serum cholesterol level, cardiac issues, T2DM, MetS, fatty liver disorders, breathing difficulty, sleep apnea, and bronchial asthma [52]. Additionally, obesity among children causes psychical, emotional, cognitive, societal or civic, compartmental, attitudinal, and communicative out-turn, e.g., increases the probability of issues associated with body appearance, self-respect, dignity, loneliness, social withdrawal, stigma, depression, inequity or chauvinism, and overall childhood obesity take at the edge of poor quality of life [53-57]. The central basis of childhood overweight and obesity is a disparity in energy between ingestion and depletion [11,58].

Objective of the study

This study emphasizes the principal causes of childhood obesity and prevention strategies.

## Review

Materials and methods

**Figure 2:**
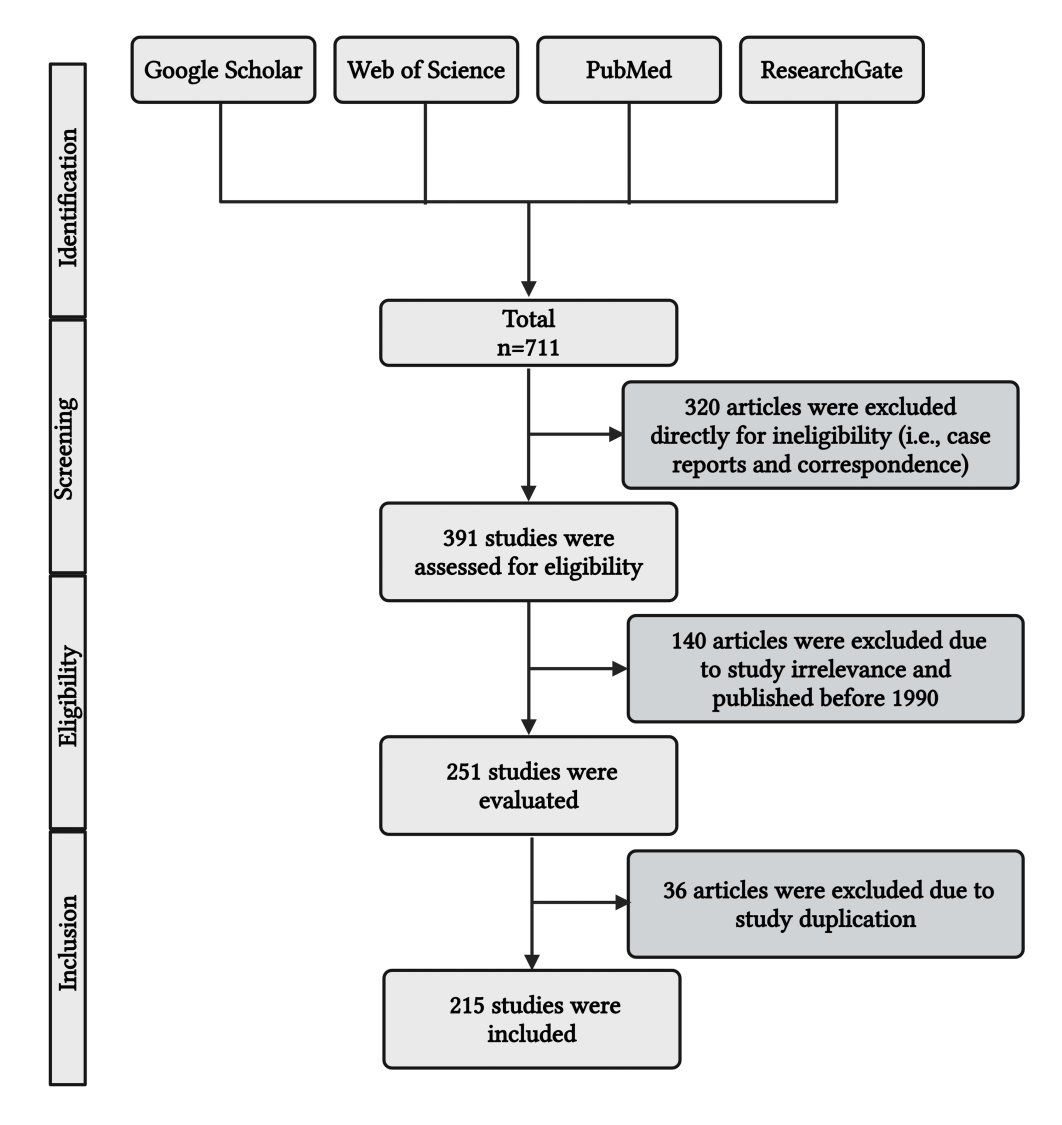
PRISMA flowchart showing the methodology of this study. PRISMA: Preferred Reporting Items for Systematic Reviews and Meta-Analyses

This article discusses how children's excessive weight gain, physical inactivity, prolonged screen time, and consumption of processed, high-calorie foods affect their well-being. The literature search used electronic archiving resources such as Web of Science, Google Scholar, PubMed, and ResearchGate (Figure 2). Again, we looked through the reference list of comparable pieces to identify further content. Keywords included "children," AND "childhood obesity, metabolic syndrome, processed foods, children's cardiovascular illnesses, screen time," AND "Physical inactivity," AND "Insulin resistance," AND "diabetes mellitus," AND "cardiovascular disease." Papers published before 2000 and printed in dialects other than English were not encompassed. The papers' appropriateness was thoroughly evaluated before their inclusion in the study. Duplicate pieces of literature were utterly discarded. Subsequently, a separate assessment and inclusion of the recommended related writings were conducted, and a supplementary conversation was conducted to find any questions.

Review of the literature

Definition of Childhood Obesity

Cleveland Clinic described that "the medical definition of childhood obesity is having a body mass index (BMI) at or above the 95th percentile for age and sex in children aged 2 years and older" [60]. Instead of traditional height versus weight charts, BMI defines people as underweight, normal, overweight, or obese. The National Institutes of Health (NIH) and the WHO use these classifications for BMI [61].

Measurement and Patterns of Adiposity in Children

Adiposity in children can be measured using various methods, including BMI, which measures weight concerning height and designates body fat proportion (kg/m^2^). BMI is applied to compute a percentile status to establish if a child is obese [62]. Waist circumference (WC) (94 and 102 cm for men and between 80 and 88 cm for women) quantifies the entire and abdominal fat intensities. It's assessed at the center between the nethermost rib and the uppermost of the hip [63-65]. Fredriksen et al. (2018) state that it is a more back-breaking job to set up a cut-off among the pediatric population with prognosticative values for disease, predominantly since many lifestyle-associated diseases do not reveal until manhood or womanhood or advanced age [65]. Skinfold thickness is an estimation of the bulkiness of the skin in diverse parts of the human body, e.g., the supra iliac, triceps, biceps, and subscapular [66,67]. Bioelectrical impedance analysis (BIA) assesses body fat proportion [68-70]. Dual-energy X-ray absorptiometry (DEXA) is another procedure for evaluating the body fat's total portion of lipids [71,72]. Computerized tomography (CT) is a process for determining the amount of body fat [73,74]. Magnetic resonance imaging (MRI) is another method for calculating body fat [75,76].

Cause of Childhood Obesity

Children's dietary habits are also associated with high body fat; multiple studies unearthed that children who eat and drink more calories containing fast food and SSBs, less and poor access to healthy foods, e.g., fruits and vegetables, had a higher tendency to be obese [77-79]. These days, developing and developed children have poor aerobic physical activity, spending considerable time in desk-bound gaming on the computer or watching television. Very rarely involved in outdoor games, e.g., football, cricket, baseball, etc. [80-82]. It is a settled issue that is associated with not having poor sleep patterns and increases the possibility of childhood obesity [83-85]. Those children who have poor quality and quantity of sleep raise the likelihood of metabolic and endocrine deviations [86], including, e.g., ﻿"decreased insulin sensitivity, decreased glucose tolerance, increased evening concentrations of cortisol, increased levels of ghrelin, decreased levels of leptin and increased hunger and appetite" [87] that trigger obesity [88,89]. Another study revealed that poor sleep patterns among children promote stress, impulsivity, unhappiness, nervousness, hostile behavior, and irrational thinking processes [90]. The children with deficient sleep additionally had compromised cognitive physiology, e.g., concluding an issue, negotiating tussle, difficulty performing mental tasks, and learning processes [91]. Current modern and urban life often causes parental stress that triggers obesity in children.

Moreover, parents remain too busy to spend less time with their children, and the scarcity of time to prepare food at home directly promotes unhealthy fast-food consumption [92,93]. Another critical issue reported is that to mitigate stress, boredom, and difficulties, children's overeating practice prevails [94,95]. Parental and sibling obesity remains a strong predictor for the development of childhood obesity. So far, several fat mass and obesity-associated (FTO) genes, e.g., *MC4R*, *LEP*, *LEPR*, *PCSK1*, and *POMC*, have been identified as instigating childhood obesity [96-99]. Wholesome, nutritious foods, e.g., fresh fruits, vegetables, nuts, legumes, etc., are often more expensive than energy-dense, high-carbohydrate, and fat-containing food. People living in low-cost areas who are primarily dependent on ready-to-eat meals in both childhood and adulthood suffer from obesity, malnutrition, and metabolic-related disorders [50,100-103]. Additionally, plenty of availability of low-cost junk food in the local area educational premises, even in developing countries, further promoted childhood obesity [104-106]. Another critical issue, especially in low-income countries, is that rapid urbanization occupies all vacant land where children used to play; speedy metropolitan cities alter lifestyle very quickly; further, high-cost outdoor games often impair playing ground activity, influencing decreased physical activity among children and promoting obesity [107-111].

The International Federation of Gynecology and Obstetrics (FIGO) has recognized NCDs as a new zone emphasizing maternal disorders [112]. Women suffer from inadequate nutritional status and low-fat reserve, especially in the pre-pregnancy period; these patients, when pregnant, need to minimize their basal metabolic rate (BMR) to support their fetus's energy requirement. This phenomenon is remarkably observed in low-income countries [113,114]. Whereas those cases start pregnancy with satisfactory nutritional status and standard body weight, BMR gradually rises throughout pregnancy [115,116]. Insufficient nutritional status among gravid ladies frequently triggers adverse impacts on unborn offspring; often, these prenatal or zygote progenesis develop IR, T2DM, and MetS later in their lives [117-119]. This is an adverse adaptation process in the in-utero environment for the survival of the fetus and frequently leads to a discrepancy concerning prenatal and postnatal life [119-122]. These nutritional insufficiencies of pregnant women usually lead to childhood obesity [123]. Multiple studies reported that living areas often do not have proper healthy food shops, particularly fresh vegetables and fruits, in an accessible distance.

Additionally, healthy foods frequently cost a lot more money for ordinary people of LMICs [124-126]. Several medicines have been reported to have a strong association with increasing weight gain and obesity [127,128]. These include antidiabetic medications (insulin, sulfonylureas, thiazolidinediones), drugs relieving epilepsy, depression, psychosis, steroid molecules containing any medicines, progestins, first-generation antihistamines, e.g., cyproheptadine, β-receptor antagonist, e.g., propranolol, and α-receptor antagonist, e.g., terazosin [6,129-136]. Alamnia et al. (2023) suggested that close observation is required when these medicines are prescribed to children to halt extreme weight gain [6].

Contribution of Imbalance in Food Intake

Children who consume more energy-dense, high-fat, low-fiber food than they need usually store the excess as body fat [137-139]. It has been reported that an additional 30 kilocalories daily, or about 2% surplus calorie intake, triggers obesity in the coming days [140]. Aggressive fast-food advertising that eating out ready-to-meal regularly causes "caloric imbalance" [141] remains a considerable contributor to childhood obesity [77,141-143].

Feeding Bottles and Cups Influence Childhood Obesity

Bisphenol A (BPA) is a chemical ingredient applied with other substances to assemble the coating for food and beverage canisters and bottles made of polyethylene or polymers, epoxy adhesives, and varnishes to prevent corrosion and extend shelf life [144]. Minute amounts of BPA frequently transfer into drinks and food from BPA-containing polycarbonate food pots [145]. Food and Drug Authority (FDA) banned BPA in manufacturing nursing or feeding bottles and children's sippy cups and training cups or beakers in 2012 [146]. Nonetheless, the fetus and infant still possess the potential possibility to be unprotected from BPA through the pregnant mother or breast milk [147,148]. Multiple prospective studies revealed fetus contact with BPA has related to adverse consequences, e.g., childhood obesity (especially among pregnant women who had high concentrations of BPA in urine) [149-151], brain developmental errors because affecting the endocrine system [152,153], cutbacks in fetal growth [154-156], and low-grade albuminuria among those children whose mother had high-level BPA urinary excretion [157-159].

Phthalates are a cluster (dioctyl phthalate, di(2-ethylhexyl) phthalate (DEHP), benzyl butyl phthalate (BBP), dibutyl phthalate, di-n-octyl phthalate (DOP), dimethyl phthalate, diisononyl phthalate (DINP)) of man-made chemical compounds utilized for making plastics more malleable, see-through, and long-lasting. Furthermore, as adhesives, phthalates are used in diluents, toiletries, cosmetics, children's toys, medical tubing, intravenous infusion bags, catheters, and building materials [160-163]. Persistent contact with phthalates harmfully influences the endocrine and other multiple organs, thereby causing adverse impacts on pregnancy outcomes, child growth and development, and reproductive systems equally among youngsters and adolescents [161,164-166]. Mariana et al. (2016) reported that chronic contact with phthalates triggers adverse health impacts, predominantly affecting reproductive and cardiovascular physiology [167].

Qian et al. (2019) [168] reported that prenatal women exposed to high-molecular-weight (HMW) phthalates possess an adverse connotation between psychomotor development index (PDI) scores observed in female children. In contrast, a positive relation was observed among boys. Researchers steadily detected that the exposure quotient of di-n-butyl phthalate (DnBP) was inversely related to PDI outcomes in all children. However, the risk index of DEHP, an HMW phthalate related to PDI scores among boys only, was assessed through cumulative risk assessment analyses. Thereby, an adverse relationship was observed between prenatal contact with mono-n-butyl phthalate (MnBP) and neonatal psychomotor progress [166]. Phthalates specifically instigate adverse impacts expressly in the prenatal and the early postnatal periods. Phthalates impede the thyroid hormone beckoning or metabolic process, affecting the neuroendocrine system. It triggers the interruption of neuronal diversity and evolution process, raising the possibility of neurocognitive ailments, e.g., psychic difficulties, low-rate psychological, cerebral, psychomotor, and intelligence quotient (IQ) developmental process, attention-deficit hyperactivity disorder (ADHD) and autistic comportments [169]. Phthalates have been banned or restricted in many countries, including the European Union (EU), especially for food packaging material, and the FDA has banned the straightforward addition of phthalates in food for the USA [164,170,171].

Food Preservatives and Additives

Currently, propionic acid (PA) is a typical food preservative, and it was recognized by the US FDA as harmless in 1984 for human consumption as an additive [172]. PA innately arises as a short-chain fatty acid (SCFA) that possesses pharmacodynamics [173] in preventing the formation of fungus [174] in diverse processed foods, including bakery products, e.g., white bread, baked glazed donuts, cookies, banana cakes, chocolate brownies, chocolate chip cookies, cheesecake, cupcakes, etc., tortillas and cheese [175,176]. PA disrupts metabolic processes by increasing hormones, e.g., glucagon, norepinephrine, etc., connected with IR, T2DM, and obesity [170]. It has been observed that obese and overweight children pass feces containing higher levels of PA [177,178]. Globally, children and adolescents from both HICs and LMICs consume processed foods [15,18,179-184].

Monosodium glutamate (MSG) is naturally found in cheese and fruit juices like grape juice, mushrooms, broccoli, and tomatoes [185]. It is also added as a taste enhancer in ﻿canned vegetables, restaurant foods, stock cubes (dried bouillon cubes), soup, ramen, gravy, stews, deli meats, condiments, sauces, savory and salty snacks, soy sauces, spices, etc. [186]. MSG regular consumers have an increased possibility of greater BMI, overweight, and obesity than those who avoid this taste modifier [187,188]. Additionally, those individuals who chronically consume MSG-containing food, in general, take higher portions of energy-dense food, e.g., high sugary food, animal protein, cholesterol, fats, and minimum eating and drinking lesser ingestions of wholesome, balanced food, e.g., vegetables, fresh foods, nuts, legumes, high fiber containing food, and magnesium [187]. MSG consumers had a higher possibility of developing MetS [189]. MetS is considered a group of disorders that raises the likelihood of long-lasting noncommunicable illnesses, such as high blood pressure, high lipid profile, atherosclerotic heart disease, stroke, and diabetes [190]. MSG interrupts leptin-mediated hypothalamus signaling alleyway, triggering the commotion of energy balance and promoting obesity [191-193]. Prolonged consumption of MSG as a food additive impairs the fetal developmental sequence and skeletal growth. Furthermore, it affects various histological and biochemical alterations necessary equally for the mother and embryonic hepatic and renal tissues, which denotes the noxious and teratogenic pharmacodynamics of MSG [194].

Trans Fats in Fast Foods

Fast foods are often rich in trans fatty acids; consequently, they increase body weight and cause obesity, especially among school-going children [195-197]. Jia et al. 2019, in their systematic review and meta-analysis, reported that junk food that characteristically comprises excessive amounts of "calories, saturated fat, trans‐fat, sugar, simple carbohydrates," raw sugar, and Na+ salt retailed at a comparatively low expense [198].

Internationally, the speedy growth of quick-service restaurant chains, even in LMICs, consequently eating and drinking ready-to-eat meals escalated considerably over the past few epochs along with the mounting obese population [199,200]. Easy accessibility, high palatability, low price, and aggressive advertising schemes have made junk food a favorite among all age groups, especially school-going children and adolescents [201,202]. The presence of junk food shops in the neighborhood and nearby schools potentially increases the risk of overweight and obesity, especially among kids and teenagers [203-205].

Physical Inactivity and Lifestyle

Physical activity and healthy eating are two aspects of healthy living that lower the risk of obesity in adults and children. Increasing energy expenditure through changes in physical activity is typically a key part of treating childhood obesity [206]. Again, physical activity improves the metabolic profile and psychological well-being of obese children [207]. Normal growth and development of muscle strength, motor skills, coordination, flexibility, and cardiovascular fitness require physical activity [208]. Regular aerobic exercise significantly lowers cardiovascular risk by improving lipid profiles, particularly in kids and teenagers with low baseline lipid and lipoprotein concentrations [209,210]. Bull et al. (2020) recommend that at least 30 minutes of cumulative moderate daily physical activity accounts for many health benefits [211].

According to the WHO, preschool-aged children should engage in at least 180 minutes of physical activity daily, while children and adolescents should achieve a minimum of 60 minutes of vigorous exercise daily [212]. Actigraph accelerometric measurements showed that less physically active children were likelier to have higher subcutaneous fat levels than more physically active children [213]. Engaging in sports for youngsters is crucial for encouraging children to be physically active and could be a means of preventing childhood obesity. In the United States, children aged 6 to 12 years participate in 5 to 6.5 hours of sport each week, which is crucial for developing a physically active and healthy lifestyle. Multiple studies explored how juvenile physical activity involvement affected children's BMI and fitness levels. Physical activity intervention considerably decreased BMI [214-216].

Addiction to Different Electronic Devices

Obesity rates rise as the prevalence of digital addiction grows. Technological instruments, particularly digital electronics, contribute to malnutrition by encouraging inactivity. This condition causes an increase in obesity in society and a variety of health issues. Children are adopting sedentary lives as they spend more time sitting down. These circumstances have set the stage for obesity to become a widespread health concern [217].

Psycho-Social Factors

The interplay between social variables and personal thoughts and behaviors, including dietary habits, stress, physical appearance concerns, nervousness, mental health issues, impulsiveness, and self-confidence, is known as the psychosocial element of obesity [217]. In addition to this growing academic pressure and competitive stress, children are frequently blamed for the increased hours of lack of activity caused by their evolving lifestyles in modern society [218]. A significant psychosocial factor in obesity has been identified as stress, and children who experience stress are more likely to engage in emotional overeating [210]. According to several studies, childhood obesity is linked to a range of psychosocial factors, including poorer standards of life, discrimination in society, mockery, decreased self-worth, and neuropsychiatric disorders [219,220].

Consequences of Childhood Obesity

Establishing solutions for identifying and managing children with overweight and obesity requires an extensive awareness of the physical and hereditary risks, social variables, and environmental factors that contribute to these health issues [221]. Furthermore, an essential contributing factor to the progression of obesity is initial exposure to an obesogenic environment, which includes the pre-pregnancy and pregnancy phases. The risk of pediatric obesity and T2DM in both children and adults is associated with maternal overweight, obesity, and prenatal excess weight gain [222].

**Figure 3:**
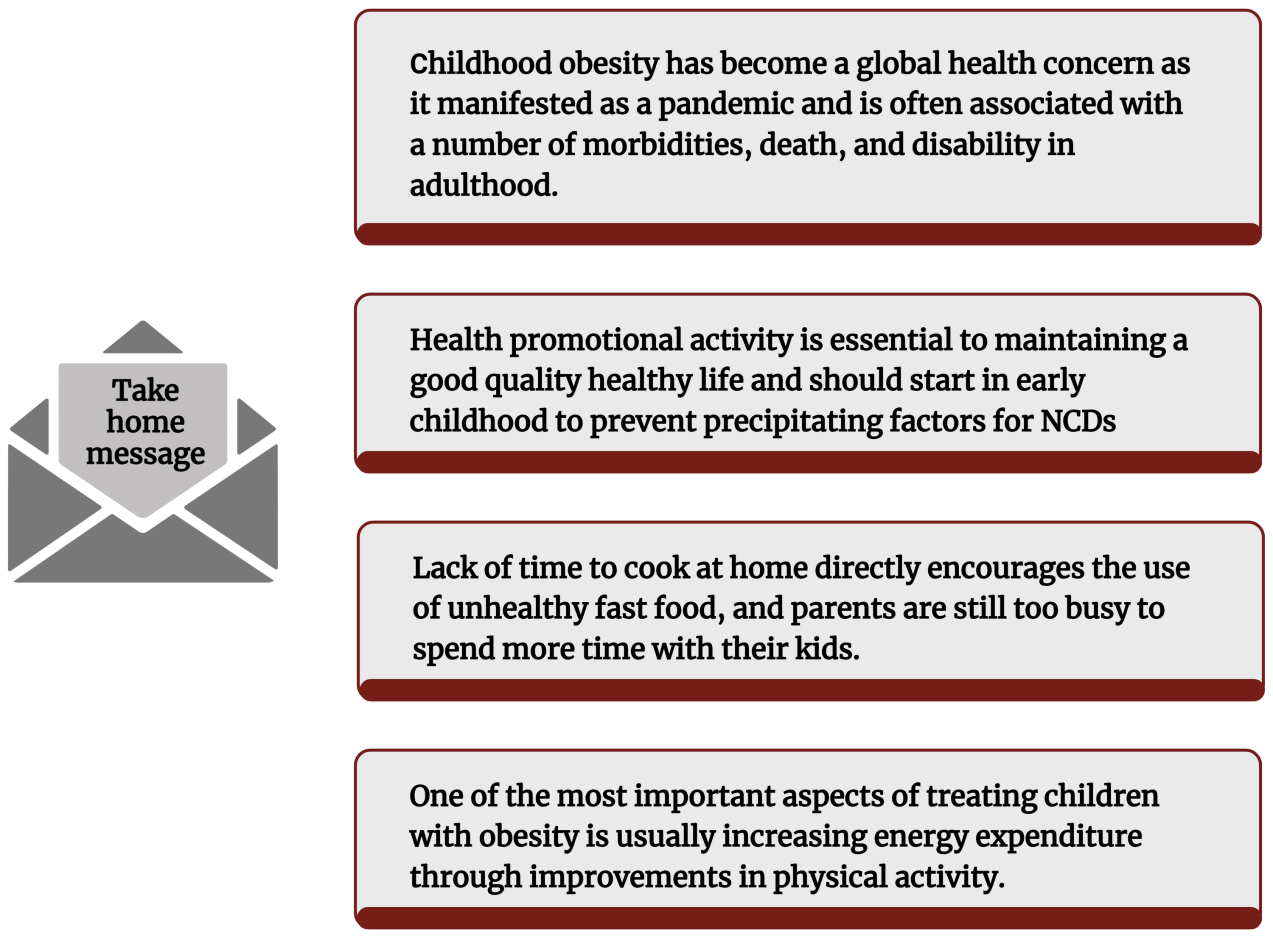
Illustration showing key finding of this paper. Notes: This figure was drawn using the premium version of BioRender (https://biorender.com/rq6mzkw) [59], which was accessed on 21 March 2025, with the license number RA281VROX8. NCDs: non-communicable diseases Illustration Credit: Susmita Sinha.

Obesity among children has been connected to several medical issues. Heart problems, elevated cholesterol levels, gallstones, fatty liver, sleep apnea, T2DM, respiratory conditions, hepatic steatosis, IR, glucose intolerance, skin disorders, irregular menstruation, impaired balance, and orthopedic issues are a few of these conditions [39,223,224]. Table 1 depicts several findings from different studies. Figure 3 illustrates the principal pieces of information in this paper.

**Table 1:** The table depicts several findings from different studies. NCDs: non-communicable diseases; T2DM: type 2 diabetes mellitus; BMI: body mass index Table Credit: Susmita Sinha.

Author’s Name	Study Type	Journal Details	Background	Result	Conclusion
Ejigu and Tiruneh [5]	Systemic review	Int J Hypertens. 2023; 2023:2199853.	Obesity and being overweight are the primary risk factors for NCDs, according to evidence. After controlling for other significant variables, the study intends to investigate the relationship between overweight/obesity and common NCDs.	Of the 9,800 attendees, 1368 had excessive cholesterol, and 2053 had hypertension. The multivariable logistic regression analysis showed a positive correlation between hypertension and overweight/obese patients.	Proper measures are required to prevent overweight and obesity by promoting physical activity, reducing inactivity, and maintaining a balanced diet to lower the risk of high blood pressure and cholesterol.
Alamnia et al. [6]	Population-based cross-sectional study	Sci Rep. 2023;13(1):21028	Improper dietary patterns are associated with several NCDs such as diabetes, hypertension, cardiovascular disorders, and obesity.	Abdominal obesity and being overweight or obese did not significantly correlate with the observed eating patterns.	Determination of a population’s main food habits can help guide nutritional strategies to lower and avoid metabolic risk factors.
Sahoo et al. [7]	Narrative review	J Family Med Prim Care. 2015;4(2):187-92	Obese children have a higher chance of being obese as adults and of developing NCDs like cardiovascular disease and diabetes earlier in life.	Childhood obesity can have serious consequences for children’s physical health, social and emotional well-being, and self-esteem.	A combined community-school-based food and physical activity strategy can effectively prevent obesity. Parents who encourage improvements in lifestyle at home can also prevent most weight issues.
Horesh et al. [12]	Systemic review	Curr Obes Rep. 2021; 10(3):301-10	The link between childhood obesity and excess morbidity and mortality has not been appropriately demonstrated yet.	Obesity in childhood and adolescence is strongly associated with cancer, diabetes, and cardiometabolic consequences in middle age.	Increased medical conditions load in middle age could result from the rising incidence of childhood and adolescent obesity, which highlights the crucial importance of early implementation for efficient treatment methods.
Yoshida and Simoes [13]	Systemic review	Curr Diab Rep. 2018; 18(6):31	Obesity and T2DM are linked to sugar-sweetened drinks (SSBs). Presently, strategies that restrict SSBs in schools, different school-based interventions, and taxation on reducing SSB intake are taken to reduce the burden of obesity.	School-based interventions and taxation on SSBs are pretty successful in opposing obesity.	Families and local communities should be included in intervention programs adapted to age, gender, culture, and language. A higher tax rate might be recommended to have a discernible impact on weight.
Banik et al. [16]	Cross-sectional study	Obes Med. 2020; 17: 100161	Adolescents attending college are overly likely to eat fast food, which raises their chance of being obese in their adult lives.	This study discovered a strong correlation between consuming fast foods and the increased risk of obesity.	To address this issue, targeted health education initiatives, dietary recommendations, and successful public awareness efforts would be ideal.
Emond et al. [17]	Prospective cohort study	Pediatr Obes. 2020;15(4):e12602	Fast food consumption is cross-sectionally linked to childhood obesity and overweight.	Over the study year, the risk of improving weight status rose linearly with each additional fast-food consumption in an average week.	An increase in weight status was linked to consuming more fast food over one year.
Machado et al. [24]	Cross-sectional study	Nutr Diabetes. 2020;10(1):39	Increased fast food consumption over a year was connected with increased weight status.	According to the multivariate regression analysis, people who consumed the most ultra-processed foods had significantly larger waist circumferences, BMIs, and probabilities of being obese and having abdominal fat than people who consumed the fewest.	The consumption of ultra-processed meals is related to a higher risk of obesity, which supports the idea that these foods may be a contributing factor to obesity in Australia.
Hagman et al. [36]	Cohort study	Int J Obes (Lond). 2019;43(10):1988-94	Childhood obesity is a significant risk factor for hypertension and blood pressure that can be ameliorated by weight loss.	A lower BMI standard deviation score (SDS) resulted in a drop in the diastolic and systolic blood pressure SDS. Again, failure to receive obesity treatment raised the chance of hypertension.	For children who are obese, losing weight is essential to both preventing and treating hypertension.
Sarwer and Polonsky [57]	Narrative review	Endocrinol Metab Clin North Am. 2016; 45 (3):677-88	Many obese people experience problems with their state of mind, self-confidence, and appearance.	Adjustments in psychosocial stability and productivity are usually linked to weight loss.	Some people who lose weight either acquire new psychosocial problems or see their pre-existing psychopathology reappear. Regardless of the method used for weight loss, people who regain their weight are as susceptible to the recurrence of undesirable psychological manifestations.
Chung [60]	Narrative review	Ann Pediatr Endocrinol Metab. 2015; 20 (3):125-9	The frequency of childhood and teenage obesity has risen in conjunction with changes in social and economic circumstances and lifestyle patterns. Childhood BMI increases are linked to adult obesity and an increased risk of several illnesses, including atherosclerosis, diabetes, and other comorbidities.	Raised BMI values in overweight children can be due to increased fat or fat-free mass. It is crucial to track alterations to physique composition since fast development coincides with changes in the endocrine system in adolescents, making it difficult to identify changes in each component.	Preventing obesity and lowering the risk of metabolic diseases throughout adolescence requires optimal development and appropriate body structure build-up.
Jakobsen et al. [77]	Systemic review and meta-analysis of observational study	Nutrients. 2023; 15 (3):764	A nutritious diet is vital for preventing childhood obesity. Food and beverages are among the risk variables that play an essential part in childhood obesity.	Intake of more sugar-sweetened beverages and fast foods increases the possibility of being overweight or obese. Also, increased meat and refined grains intake was associated with an increased risk of overweight/obesity.	The main dietary factors for overweight/obesity were found to be a higher consumption of fast food and sugar-sweetened beverages.
Scaglioni et al. [78]	Systemic review	Nutrients. 2018;10(6):706	Knowledge of the mechanisms behind food behaviors may help pediatricians promote the development of healthy eating practices throughout the child population.	The most important factors influencing a child’s eating habits and food preferences are its parents' feeding practices and eating habits. So, parents should introduce their children to various healthy dietary options.	Several elements affect eating patterns; they cannot be considered in isolation since they interact. The family structure that surrounds a child’s domestic life will play an active role in creating and promoting behaviors that will last his or her entire life.
Sivasubramanian et al. [80]	Experimental research methodology	Bioinformation. 2022;18(9):791-4	Children can lead active and satisfying lives in a natural learning environment. In addition to the importance of physical activity for kids’ health and well-being, it has also been demonstrated that letting kids spend time outside and doing play-based activities helps them develop emotional and social resilience.	This study found that 75% of children lacked information about outdoor games. Also, children’s knowledge of outdoor games is strongly connected with their parents’ educational standing. Knowledgeable parents identify the importance of outdoor activities for their children’s health.	Effective strategies are needed to educate youngsters on the benefits and drawbacks of outdoor activities.
Paduano et al. [81]	Cross-sectional study	Int J Environ Res Public Health. 2021;18(6):3221	Obese children already have specific physical and psychological issues, which often get worse as they get older. In addition, kids who are obese have a higher chance of becoming obese adults and are more susceptible to several chronic illnesses.	63% of children spend two or more hours daily in sedentary pursuits. Playing video games and using tablets/personal computers/mobile phones have the most significant impact on childhood obesity. Again, higher levels of parental education are a protective factor for preventing a sedentary lifestyle.	Interventions are required to extend physical activity time and encourage safe digital media use by the whole family, reaching all parents regardless of their ethnicity or level of education.
Ruan et al. [85]	Meta-analysis	Sci Rep. 2015; 5:16160	Sleep duration is thought to have a crucial influence on the development of obesity in children.	Children with the shortest sleep durations were 76% more prone to be obese, and they tend to gain larger yearly BMI.	The incidence of overweight and obesity in children and adolescents is inversely and longitudinally correlated with sleep duration.
Panera et al. [98]	Systemic review	Front Endocrinol (Lausanne). 2022; 13: 1006008	The failure of preventive programs and the typical inefficiency of current medicines led to several studies that have revealed some pertinent components of the genetic and epigenetic inheritable profiles of obesity.	Independent of the transfer of a solely genetic predisposition, maternal obesity and overnutrition during pregnancy and lactation appear to be connected with many illnesses in the offspring. In addition, reprogramming the epigenetic architecture of cells can occur even if the mother or father gametes are directly exposed to environmental influences before conception.	An overall summary of how heritable genetic and epigenetic factors contribute to children’s vulnerability to obesity, with a focus on the mother-child relationship.
Singh et al. [128]	Systemic review	Obesity (Silver Spring). 2021;29(2):265-73	Weight gain is caused mainly by medications in many cases, and the most significant drug classes are frequently linked to weight gain as an adverse effect.	The clinician can choose the best medication for a patient using their pharmacogenomic profile to determine which medications are most likely to produce weight gain as a side effect.	The function of pharmacogenomics in anti-obesity drugs requires further research.
Vernarelli et al. [138]	Cross-sectional study	Eur J Nutr. 2018; 57 (1):351-61	It is unclear if the percentage of the diet that consists of low energy density (ED) items is correlated with weight status, despite recent public health messages advising consumers to reduce dietary ED for weight management.	Compared to males classified as obese, lean males were found to consume a more substantial percentage of total calories from very low and low energy density items. Also, lean females reported consuming 7.8% of their total energy from very low-energy dietary foods of 24%.	Lower BMI and waist circumference are linked to higher percentages of food weight and energy intake that come from extremely low and low ED diets.
Wang and Qian [161]	Narrative review	Healthcare (Basel). 2021; 9(5):603	Phthalates are a group of commonly applied chemical substances that have been shown to adversely affect the endocrine system and harm the health of individuals.	Exposure to phthalates is linked to altered proline and arginine metabolism, which causes children to become obese.	This article outlines the harmful effects of phthalates on human health, examines the toxicity mechanism, evaluates the dangers, and then offers workable solutions to lower public exposure to phthalates.
Adler et al. [172]	Randomized controlled trial (RCT)	BMJ Open Diabetes Res Care. 2021;9(1):e002336	The effects of exogenous propionic acid on glucose metabolism are not entirely known.	Glucagon, norepinephrine, and endogenous glucose synthesis are all markedly increased by propionic acid.	The insulin counterregulatory hormonal network is inappropriately activated when propionic acid is taken orally. Also, this improper activation emphasized propionic acid as a metabolic disruptor.
Jia et al. [198]	Meta-analysis	Obes Rev. 2021;22 Suppl 1 (Suppl 1):e12944	It is believed that having too many fast-food restaurants in the neighborhood increases the risk of juvenile obesity by discouraging good eating habits and exposing children to unhealthy food places, leading to the compensatory consumption of unhealthy food options.	Of 39 studies, 17 found that fast-food restaurant access considerably correlates with overweight/obesity. However, the majority of the research found a link between obesity and increased consumption of fast foods.	A relatively ambiguous association was found between fast-food restaurant access and obesity.
Keane et al. [206]	Cross-sectional study	Pediatr Exerc Sci. 2017;29(3):408-18	Public health strategies worldwide focus on changeable lifestyle choices.	This study found obesity in 23.7% of all children. Again, independent of overall leisure activities, the likelihood of being overweight or obese was negatively correlated with the amount of time spent at moderate to vigorous physical activity (MVPA). Regardless of physical activity, screen time was linked to a higher chance of becoming overweight or obese.	Screen time and physical activity recommendations were not reached by many kids, indicating the need for population-based interventions.
Sagar and Gupta [220]	Narrative review	Indian J Pediatr. 2018;85(7):554-9	Children who are obese face a variety of psychosocial issues that have a substantial impact on their well-being and quality of life.	Psychological factors such as anxiety, stress, depression, eating disorders, low self-esteem, and issues with body shape are linked to childhood obesity.	Cognitive behavioral therapy (CBT) techniques in conjunction with lifestyle interventions and parent involvement have been suggested.
Faienza et al. [223]	Narrative review	World J Pediatr. 2020;16(5):438-45	The prevalence of childhood obesity has grown over the last three decades, and the trend constitutes a concerning epidemic worldwide.	In people whose BMI rose during adolescence, there was a connection between an elevated risk of cardiovascular events and all-cause death. Depending on the situation, the population under study, and the ethnicity, the frequency of non-alcoholic fatty live disease in kids and teens and in obese kids varies from 6% to 38%.	According to this study, the most effective therapy strategies for children to reduce the risk of obesity and lessen the incidence of cardiovascular disease and diabetes in adulthood are enhancing physical activity and improving food behavior.

Future Research Recommendation

**Figure 4:**
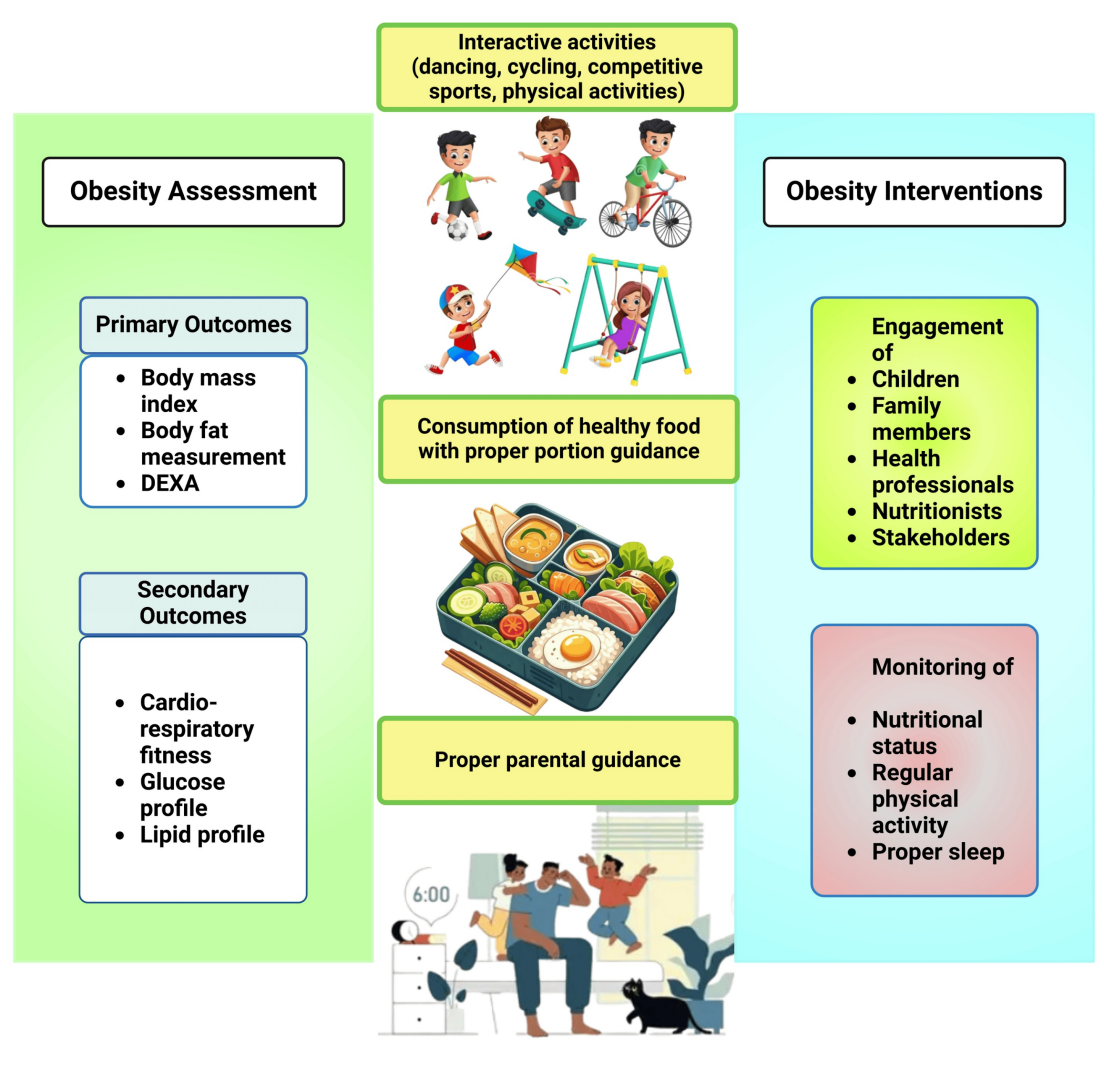
Schematic diagram showing interventions that can be taken for obese children. Notes: This figure was drawn using the premium version of BioRender [59] (https://biorender.com/45b6mam), which was accessed on 21 March 2025, with the license number ZU281U2W0J. DEXA: dual-energy X-ray absorptiometry Illustration Credit: Susmita Sinha.

Standardizing indicators and definitions for obesity-related measurements would help better compare obesity prevalence and program efficiency within and between nations (Figure 4). Furthermore, it would create and manage a database for population health-based plans. Obesity management and prevention research funds should involve rigorous methods considering sample size, study design, and validity.

Limitations of the Study

More clinical trials with sizable sample sizes across a range of ethnic communities are needed, and the review's findings must be applicable worldwide. More longitudinal research and systematic reviews must also be conducted to evaluate the relationship between obesity and other comorbidities, including diabetes, IR, cardiorespiratory illnesses, depression, and low self-esteem. Furthermore, it is necessary to investigate the psychosocial and systemic aspects contributing to childhood obesity.

## Conclusions

Obesity in children is a known risk factor for a variety of serious health issues. The contributing factors in children must be changed to mitigate or avoid any harm to the target organ. Therefore, preventing childhood obesity is a top public health objective for the betterment of society as well as for the eventual well-being of people. In addition, children's home experiences with good food, exercise, and nutrition can impact their lives long-term. Approaches to change behavior at the individual level are now urgently required due to the severe long-term effects of obesity, especially in young children.
